# Interhemispheric connectivity during bimanual isometric force generation

**DOI:** 10.1152/jn.00876.2015

**Published:** 2015-11-04

**Authors:** Jinyi Long, Toshiki Tazoe, Demetris S. Soteropoulos, Monica A. Perez

**Affiliations:** ^1^Department of Neurological Surgery, The Miami Project to Cure Paralysis, University of Miami, Miami, Florida; and; ^2^Institute of Neuroscience, Newcastle University Medical School, Newcastle upon Tyne, United Kingdom

**Keywords:** bilateral control, voluntary movement, cortico-cortical coupling, ipsilateral cortical silent period, bilateral force

## Abstract

Interhemispheric interactions through the corpus callosum play an important role in the control of bimanual forces. However, the extent to which physiological connections between primary motor cortices are modulated during increasing levels of bimanual force generation in intact humans remains poorly understood. Here we studied coherence between electroencephalographic (EEG) signals and the ipsilateral cortical silent period (iSP), two well-known measures of interhemispheric connectivity between motor cortices, during unilateral and bilateral 10%, 40%, and 70% of maximal isometric voluntary contraction (MVC) into index finger abduction. We found that EEG-EEG coherence in the alpha frequency band decreased while the iSP area increased during bilateral compared with unilateral 40% and 70% but not 10% of MVC. Decreases in coherence in the alpha frequency band correlated with increases in the iSP area, and subjects who showed this inverse relation were able to maintain more steady bilateral muscle contractions. To further examine the relationship between the iSP and coherence we electrically stimulated the ulnar nerve at the wrist at the alpha frequency. Electrical stimulation increased coherence in the alpha frequency band and decreased the iSP area during bilateral 70% of MVC. Altogether, our findings demonstrate an inverse relation between alpha oscillations and the iSP during strong levels of bimanual force generation. We suggest that interactions between neural pathways mediating alpha oscillatory activity and transcallosal inhibition between motor cortices might contribute to the steadiness of strong bilateral isometric muscle contractions in intact humans.

animal studies showed that static bimanual force generation involves activity-dependent adaptations in both primary motor cortices (Murthy and Fetz 1996; Soteropoulos et al. 2011). In agreement, electrophysiological studies in humans using transcranial magnetic stimulation (TMS) demonstrated that isometric bilateral force generation changes the excitability of corticospinal and cortico-cortical projections compared with unilateral force (Sohn et al. 2003; Soteropoulos and Perez 2011; Yedimenko and Perez 2010). Neuroimaging (Theorin and Johansson 2007) and cortex-muscle coherence (Kilner et al. 2003; Perez et al. 2012) studies also revealed that both motor cortices showed distinct changes in activity during bimanual compared with unilateral isometric forces. Although it is well accepted that interhemispheric interactions between motor cortices through the corpus callosum play an important role in the control of bimanual forces (Carson 2005; Diedrichsen et al. 2003; Giovannelli et al. 2009; Perez et al. 2014; Tazoe et al. 2013), the extent to which physiological connections between motor cortices are modulated during increasing levels of bimanual force generation remains poorly understood.

The combination of electroencephalographic (EEG) recordings and physiological circuits tested by TMS has provided a means to examine activity in overlapping neuronal populations (Farzan et al. 2013; Paus et al. 2001). We tested two well-known measures of interhemispheric connectivity, coherence between EEG signals in sensorimotor cortices (Andrew and Pfurtscheller 1996; Serrien et al. 2003) and the ipsilateral cortical silent period (iSP; Ferbert et al. 1992). EEG-EEG coherence (Pfurtscheller and Lopes Da Silva 1999) and the iSP (Boroojerdi et al. 1996) are thought to be mediated by long axons passing through the corpus callosum, with a net result coming from coupling between motor cortices and other cortical areas and their interaction with excitatory and inhibitory cortical circuits. EEG-EEG coherence between sensorimotor cortices in the alpha frequency band and the iSP have been associated with the size and integrity of the corpus callosum (Meyer et al. 1995; Okumura et al. 2013; Stancak et al. 2002; Teipel et al. 2009). Also, studies showed that oscillations between sensorimotor cortices in the alpha frequency band (Abdul-latif et al. 2004; Svoboda et al. 2002) and the iSP (Fling and Seidler 2012; Soteropoulos and Perez 2011) are sensitive to detect changes during strong levels of force generation. Thus we hypothesized that increasing levels of bilateral isometric forces will change interhemispheric EEG-EEG coherence in the alpha frequency band in association with the iSP. Because alpha oscillatory activity (Haegens et al. 2011; Mehrkanoon et al. 2014) and the iSP (Tazoe and Perez 2013) might play a role in error corrections and in suppressing task-irrelevant activity, we also expected that modulation of these physiological interactions will relate to the ability to maintain steady muscle contractions. Evidence showed that interhemispheric interactions between motor cortices can be modulated by somatosensory inputs (Swayne et al. 2006). Therefore, to further examine the relationship between coherence and the iSP during increasing levels of bimanual forces we used electrical stimulation of a peripheral nerve because alpha oscillations (Budini et al. 2014) and interhemispheric inhibition measured by TMS (Tsutsumi et al. 2012) can be modulated by similar afferent inputs.

## METHODS

### 

#### Subjects.

Sixteen healthy volunteers (8 men, 8 women; 25.6 ± 1.3 yr old, 14 right handed) were included in the study. All subjects gave their informed consent to the experimental procedures, which were approved by the local ethics committee at the University of Miami. The study was performed according to the guidelines established in the Declaration of Helsinki. Previous studies reported that there is between-subject variance in the magnitude of coherence and iSP measurements (Mima et al. 2000; Perez et al. 2012, 2014; Ushiyama et al. 2011). Therefore, subjects were preselected out of a total of 25 individuals who were screened to ensure that they showed interhemispheric EEG-EEG coherence between sensorimotor cortices at rest and a visible iSP in the first dorsal interosseous (FDI) muscle during unilateral 10% of maximal isometric voluntary contraction (MVC). This allowed us to measure changes in EEG-EEG coherence and the iSP during our different experimental conditions.

#### Recordings.

**Fig. 1.**
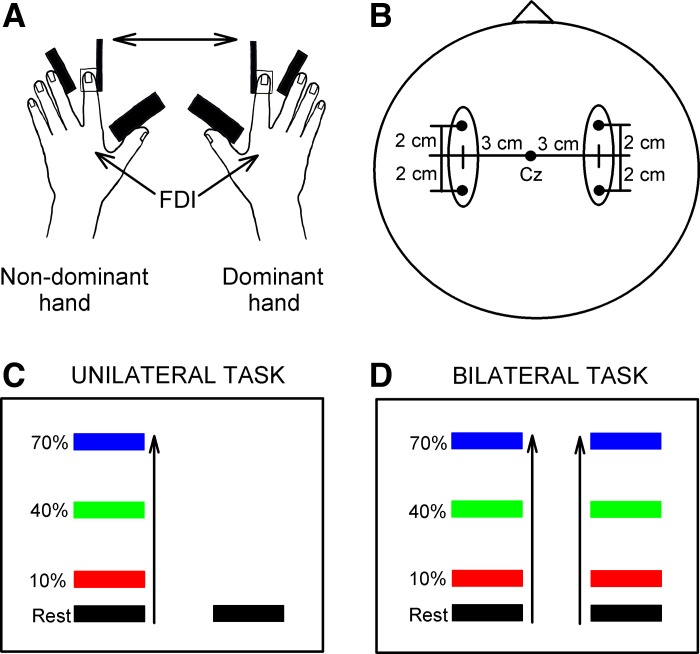
Experimental setup. *A*: schematic of the experimental setup showing the posture of both hands during testing. FDI, first dorsal interosseous. *B*: electrode positions for electroencephalographic (EEG) recordings. *C* and *D*: diagrams showing the visual display presented to all subjects during testing of unilateral and bilateral isometric index finger abduction. Subjects were instructed to perform 10%, 40%, and 70% of maximal isometric voluntary contraction (MVC) with the index finger into abduction with the nondominant hand while the contralateral dominant hand remained at rest (unilateral trials, *C*) or performed 10%, 40%, and 70% of MVC (bilateral trials, *D*). Colored bars represent the targets to which subjects needed to move a cursor. Distance between the bars represents the magnitude of force required to accomplish each task, normalized to the index finger abduction MVC determined in each participant.

Electromyographic (EMG) activity was recorded bilaterally from FDI muscles by surface electrodes (Ag-AgCl; 10-mm diameter) secured to the skin over the belly of each muscle. For measurements of coherence, EEG activity was recorded from sensorimotor cortices bilaterally with pairs of adhesive Ag-AgCl electrodes positioned 3 cm lateral and 2 cm anterior or posterior to the vertex (Perez et al. 2012; Fig. 1*B*). These locations correspond to regions between C3 and C4 areas and Cz in the 10–20 system. EEG from each side was derived from a differential recording between the electrode pair on that side; the anterior electrode was connected to the noninverting input of the amplifier. Signals were amplified and filtered (EMG: gain 500-2,000, band pass 30 Hz–2 kHz; EEG: gain 50K, band pass 3 Hz–2 kHz). EMG together with force signals were sampled at 1,000 Hz, while EEG was sampled with 5,000 Hz (Spike2 and Signal software, CED). We examined motor output steadiness by measuring the coefficient of variation of the rectified EMG and force amplitude (Graziadio et al. 2010) in the FDI muscle during unilateral and bilateral 10%, 40%, and 70% of MVC. The stability indexes of EMG (sEMG) and force (sForce) were estimated as follows:

$$\text{sEMG}=1-\frac{\text{SD}\left({\text{EMG}}_{\text{Rectified}}\right)}{\text{mean}\left({\text{EMG}}_{\text{Rectified}}\right)}$$

$$\text{sForce}=1-\frac{\text{SD}\left(\text{Force}\right)}{\text{mean}\left(\text{Force}\right)}$$

#### Experimental paradigm.

Subjects were seated with both arms flexed at the elbow by 90° with the forearm pronated and the wrist restrained by straps. The left and right index fingers were attached to custom two-axis load cells, which measured the forces exerted by the subject (Fig. 1*A*). At the start of the experiment, subjects performed two or three brief MVCs (3–5 s) with the index finger into abduction, separated by 60 s. The maximal forces were used to set targets for subsequent submaximal contractions. During maximal contractions subjects were verbally encouraged to perform maximally and visual feedback was provided (Gandevia 2001). All subjects participated in two testing sessions. In one session we assessed EEG-EEG coherence between sensorimotor cortices, and in the other session we assessed the iSP at rest and during unilateral and bilateral index finger abduction at 10%, 40%, and 70% of MVC in a randomized order (Fig. 1, *C* and *D*). Custom software was written to acquire signals from load cells to display visual feedback corresponding to 10%, 40%, and 70% of MVC in real time (LabVIEW). Subjects were instructed to perform unilateral or bilateral forces by controlling one or two cursors on a computer monitor to a target line displaying the force target. Additional verbal feedback was provided to the subjects to ensure that both hands performed the correct task at all times. Note that unilateral index finger abduction was performed with the nondominant hand. Four sets were tested with 5- to 10-min intervals. Each set consisted of 10 trials per condition; each trial lasted 4 s, with 15 s of rest between trials. Thus subjects performed 40 trials in each condition in a randomized order.

#### EEG-EEG coherence.

**Fig. 2.**
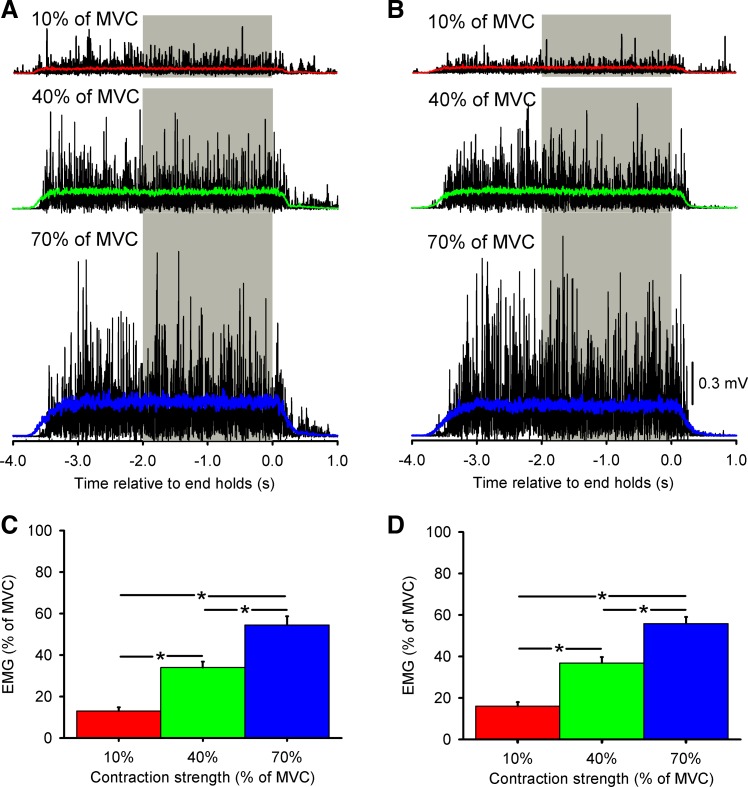
Electromyographic (EMG) recordings. *A* and *B*: in a single representative subject, mean rectified EMG activity in the nondominant (*A*) and dominant (*B*) hand performing bilateral fingers abduction of 10% (red), 40% (green), and 70% (blue) of MVC is shown. Gray bars mark the region over which coherence analysis was performed. *C* and *D*: group data (*n* = 16) showing mean rectified EMG activity (expressed as % of MVC) during bilateral index finger abduction in the nondominant hand (*C*) and the dominant hand (*D*). Error bars indicate SEs. *P < 0.05.

As the iSP measures interhemispheric inhibition with directionality, we examine functional coupling between sensorimotor cortices by using EEG-EEG directed coherence (Kamiński and Blinowska 1991). We measured EEG-EEG directed coherence from the dominant to the nondominant hemisphere in the alpha (8–13 Hz) and beta (13–30 Hz) frequency bands across conditions. At rest, EEG-EEG coherence in the alpha frequency band (mean across subjects = 0.13 ± 0.01, range across subjects: 0.049–0.23) was larger than coherence in the beta frequency band (mean across subjects = 0.067 ± 0.005, range across subjects: 0.038–0.11; *P* < 0.001). Similarly, normalized resting EEG power in the nondominant (*P* < 0.01) and dominant (*P* < 0.01) hemispheres was larger in the alpha compared with the beta frequency band. Since individuals performed isometric unilateral and bilateral index finger abduction for 4 s, we confined the analysis to the last 2 s of the hold phase of the task (see shaded area in Fig. 2*A*). The EEG data were also visually inspected to reject trials with eye movements or excessive muscle artifacts, and data were downsampled to 500 Hz. A total of 5.4 ± 4.7 trials in which eye movements or excessive muscle artifacts were detected in EEG recording were excluded from further analysis. EEG-EEG directed coherence was also measured from the nondominant to the dominant side to make comparisons across hemispheres as needed.

For EEG-EEG directed coherence calculation, two nonoverlapping 500-time point segments (corresponding to a time period of 2 s extending back from the end of trial) were taken from each trial and processed by an autoregressive (AR) model with its order selected by the Bayesian information criterion (Schwarz 1978). As in Witham et al. (2011), directed coherence (DC) was calculated by using the averaged AR model coefficient and normalized as suggested by Geweke (1982):

$${\text{DC}}_{d\to n}\left(f\right)=\frac{\left|H_{nd}\left(f\right)H_{nd}^{*}\left(f\right)C_{dd}\right|}{\left|H_{nd}\left(f\right)H_{nd}^{*}C_{dd}+H_{nn}\left(f\right)H_{nn}^{*}\left(f\right)C_{nn}\right|}$$

where *H*_*nd*_ and *H*_*nn*_ are the directional transfer function representing the causal influence of signal *d* on signal *n* and signal *n* on itself, respectively, *C*_*kk*_ (i.e., *k* = *d* or *n*) is the covariance of the noise innovations of signal *k* in the AR model, and * denotes complex conjugation. Directed coherence was estimated for each subject in each condition at frequency bands between 8 and 13 Hz and between 13 and 30 Hz. The significance level for the directed coherence was calculated as follows (Baker et al. 2006):

$$Z=1-0.05^{\left(1/L-1\right)}$$

where *L* is the total number of nonoverlapping sections and the directed coherence was considered significant (*P* < 0.05) if it was greater than *Z*. Several bins would be expected to be above the significance limit by chance. Therefore, the significant effect of the directed coherence was examined by using the binomial distribution to estimate the minimum number of frequency bins required to above the significance level (Witham et al. 2011). In this case, the directed coherence was considered significant if the total number of points above the significance level was more than 2 and 4 in the alpha and beta frequency bands separately. EEG-EEG coherence was not present at lower 1–2 Hz (coherence was lower than *Z*, *P* < 0.05) and higher 500–510 Hz (coherence was lower than *Z*, *P* < 0.05) frequency bands. EEG power was normalized to the total power (summed over the alpha and beta frequencies) obtained at rest. This allowed an estimate of the proportion of power contributed by a given frequency band; fixing the reference as the rest condition made changes in power easier to interpret. Mean EEG-EEG directed coherence at each frequency band at each condition was expressed as percentage of the coherence measured at rest in each subject.

#### TMS.

TMS pulses were delivered from a Magstim Rapid^2^ stimulator (Magstim) through a figure-eight coil with its handle pointing backward ∼45° away from the midline. During testing the TMS coil was held to the head of the subject with a custom coil holder, with the head secured with straps against a headrest to restrict movements. TMS measurements included resting motor threshold (RMT) and the iSP. The RMT was defined as the minimum intensity that evoked motor evoked potentials (MEPs) of at least 50 μV in peak-to-peak amplitude in at least half of 10 consecutive trials in the relaxed FDI (Rothwell et al. 1999).

#### iSP.

The iSP was measured with a previously standardized method (Trompetto et al. 2004). It was recorded in the nondominant FDI while the dominant motor cortex was stimulated when subjects performed unilateral and bilateral index finger abduction at 10%, 40%, and 70% of MVC. At the start of each experiment, the intensity of TMS was adjusted to produce a visible iSP without a previous facilitation. Based on previous literature, we started the test with intensities 10% or 20% above the RMT. If the iSP was unclear, the stimulus intensity was increased in small steps until the iSP was present without evoking a short-latency facilitation. TMS was applied at the same intensity in all conditions tested in each subject (118.7 ± 4.3% of RMT) over the dominant motor cortex. iSP onset and offset were defined as the time point when the EMG dropped below the mean (minimal duration of 10 ms) and the time point when the EMG returned through this level, respectively. The area of the iSP was calculated with the following formula: [iSP area = (mean EMG) × (iSP duration) − (au_iSP)], where mean EMG is the mean amplitude of rectified EMG for 100 ms of prestimulus period and au_iSP is the area under the rectified iSP. The iSP area was normalized against the level of contraction [iSP area normalized to contraction = iSP area/(mean EMG × mean duration of iSP)]. Mean duration of iSP was obtained from all conditions in all subjects. During each contraction, TMS was delivered three times at 1-s intervals to give a total of 30 trials of each condition. During unilateral contractions the iSP latency (10% = 33.5 ± 3.7 ms, 40% = 34.6 ± 4.2 ms, and 70% = 33.6 ± 3.1 ms; *P* = 0.5) and duration (10% = 26.9 ± 5.1 ms, 40% = 27.4 ± 5.2 ms, and 70% = 28.6 ± 6.6 ms; *P* = 0.6) were similar across force levels, whereas the iSP area during unilateral 70% of MVC was increased compared with 40% and 10% of MVC (10% = 40.8 ± 17.8%, 40% = 42.5 ± 12.7%, and 70% = 50.0 ± 14.3%; *P* = 0.01). In additional experiments (*n* = 7), the iSP was measured in the dominant index finger while the nondominant motor cortex was stimulated (TMS intensity 116.7 ± 15.8% of RMT) when subjects performed unilateral and bilateral index finger abduction at 10%, 40%, and 70% of MVC.

#### Effects of peripheral nerve stimulation on EEG-EEG coherence and iSP.

EEG-EEG coherence between sensorimotor cortices and the iSP in the nondominant FDI muscle were measured with and without a preceding train of electrical pulses given to the dominant side ulnar nerve at the wrist at 8 Hz and 30 Hz at rest and during bilateral isometric contraction at 70% of MVC (*n* = 10). This force level was chosen since the modulation of coherence in the alpha frequency band and the iSP area was stronger at this force level. The intensity used for electrical stimulation was defined as the minimum intensity needed to evoke a motor response of at least 50 μV in peak-to-peak amplitude in at least 5 of 10 consecutive trials in the relaxed FDI (4.8 ± 1.2 mA). The last electrical pulse was given 25 ms before the TMS pulse given to elicit the iSP. First, we tested the effect of stimulation at 8 Hz and 30 Hz at rest on EEG-EEG coherence in both frequency bands. For this, we applied 8 or 27 pulses in the last second of each frame in a total of 30 frames (with 240 pulses in total at 8 Hz and 810 pulses in total at 30 Hz). Note that EEG-EEG coherence was measured at intervals between the electrical pulses' stimulus artifacts. EEG traces were visually inspected, and data 5 ms before and after each stimulus artifact were removed from the analysis. A total of 925 ms was extracted when 8 pulses were applied per frame, and a total of 735 ms was extracted when 27 pulses were applied per frame. Time points for coherence analysis were matched by analyzing coherence during 735 ms in each frame at each frequency band. Later, the iSP was tested, with the same methodology described above, during bilateral 70% of MVC alone or preceded by a train of electrical pulses at 8 Hz (240 pulses) and 30 Hz (810 pulses) in a randomized manner. We also stimulated the ulnar nerve at 8 Hz but increased the number of pulses (8 pulses/frame in 101 frames, total 808 pulses) to match the number of pulses given at 30 Hz.

#### Statistical analysis.

Two-way repeated-measures ANOVAs were performed to determine the effect of FORCE (10%, 40%, 70% of MVC) and CONDITION (unilateral, bilateral) on mean alpha and beta EEG-EEG coherence and mean normalized EEG power. The same analysis was performed to determine the effect of HAND (dominant, nondominant) and FORCE on mean rectified EMG activity, sEMG, and sForce using the resting condition in the comparisons as needed. One-way repeated-measures ANOVAs were completed to examine the effect of FORCE on mean alpha and beta EEG-EEG coherence and mean normalized EEG power during unilateral contractions and at each contraction level and also to examine the effect of FORCE on the onset, duration, and iSP area. The same analysis was used to determine the effect of STIMULATION (8 Hz, 30 Hz, no stimulation) on mean alpha and beta EEG-EEG coherence at rest and the iSP area during bilateral index finger abduction at 70% of MVC. A post hoc Tukey test was used to test for significant comparisons. In addition, two-way repeated-measures ANOVAs were performed to determine the effect of FORCE and SIDE (dominant to nondominant, nondominant to dominant) during bilateral trials on alpha and beta EEG-EEG coherence and the iSP. Pearson correlation analysis was used as needed, Bonferroni corrected for multiple comparisons. To further examine the relationship between physiological measures and motor performance, multiple regression analyses were conducted. At each force level, we used changes in sEMG across conditions as the dependent variable and changes in iSP and EEG-EEG coherence across conditions as independent variables. Significant predictions on estimated regression were determined only when variance inflation factors were <5. Significance was set at *P* < 0.05, and group data are presented as means ± SD in the text.

## RESULTS

### 

#### EMG.

Figure 2, *A* and *B*, illustrate data from a single representative subject during bilateral index finger voluntary contraction of the FDI muscle. In this subject, the mean rectified EMG activity increased in the nondominant (Fig. 2*A*) and dominant (Fig. 2*B*) hand while performing 10%, 40%, and 70% of MVC; the gray bars show the region over which the coherence analysis was completed. During unilateral contractions, we found an effect of FORCE [*F*_(2,15)_ = 188.2, *P* < 0.001] on mean rectified FDI EMG activity. Post hoc testing showed that mean rectified EMG activity increased during 40% and 70% compared with 10% of MVC (10% = 13.9 ± 6.7, 40% = 34.7 ± 11.1, 70% = 53.5 ± 11.3; *P* < 0.001). Mean rectified EMG activity was also increased at 70% compared with 40% of MVC (*P* < 0.001). We also found an effect of FORCE [*F*_(2,15)_ = 146.2, *P* < 0.001] but not HAND [*F*_(1,15)_ = 0.3, *P* = 0.6] or their interaction [*F*_(2,30)_ = 0.9, *P* = 0.4] on mean rectified FDI EMG activity during bilateral contractions [nondominant hand: 10% = 12.9 ± 7.3, 40% = 33.9 ± 11.4, 70% = 54.5 ± 16.7% of MVC, *P* < 0.001 (Fig. 2*C*); dominant hand: 10% = 15.9 ± 7.9, 40% = 36.8 ± 11.6, 70% = 55.8 ± 12.8% of MVC, *P* < 0.001 (Fig. 2*D*)] hand. EMG activity was also larger during 70% compared with 40% of MVC in both hands (*P* < 0.001).

To examine motor output steadiness we measured the sEMG and sForce across conditions. We found an effect of FORCE [*F*_(2,15)_ = 3.4, *P* = 0.04], CONDITION [*F*_(1,15)_ = 13.4, *P* = 0.002], and their interaction [*F*_(2,30)_ = 3.7, *P* = 0.03] on sEMG during bilateral compared with unilateral contractions. Here, the sEMG decreased during bilateral (10% = 0.77 ± 0.006, 40% = 0.79 ± 0.006, 70% = 0.81 ± 0.005) compared with unilateral (10% = 0.77 ± 0.007, 40% = 0.76 ± 0.01, 70% = 0.78 ± 0.006) contractions at 40% and 70% (*P* < 0.001) but not 10% (*P* = 0.2) of MVC. No changes in sEMG were observed between 70% and 40% of MVC (*P* = 0.3). Similarly, sForce decreased during bilateral (10% = 0.86 ± 0.02, 40% = 0.88 ± 0.01, 70% = 0.91 ± 0.01) compared with unilateral (10% = 0.87 ± 0.012, 40% = 0.91 ± 0.01, 70% = 0.94 ± 0.01) contractions at 40% (*P* = 0.01) and 70% (*P* = 0.002) but not 10% (*P* = 0.6) of MVC.

#### EEG-EEG coherence.

**Fig. 3.**
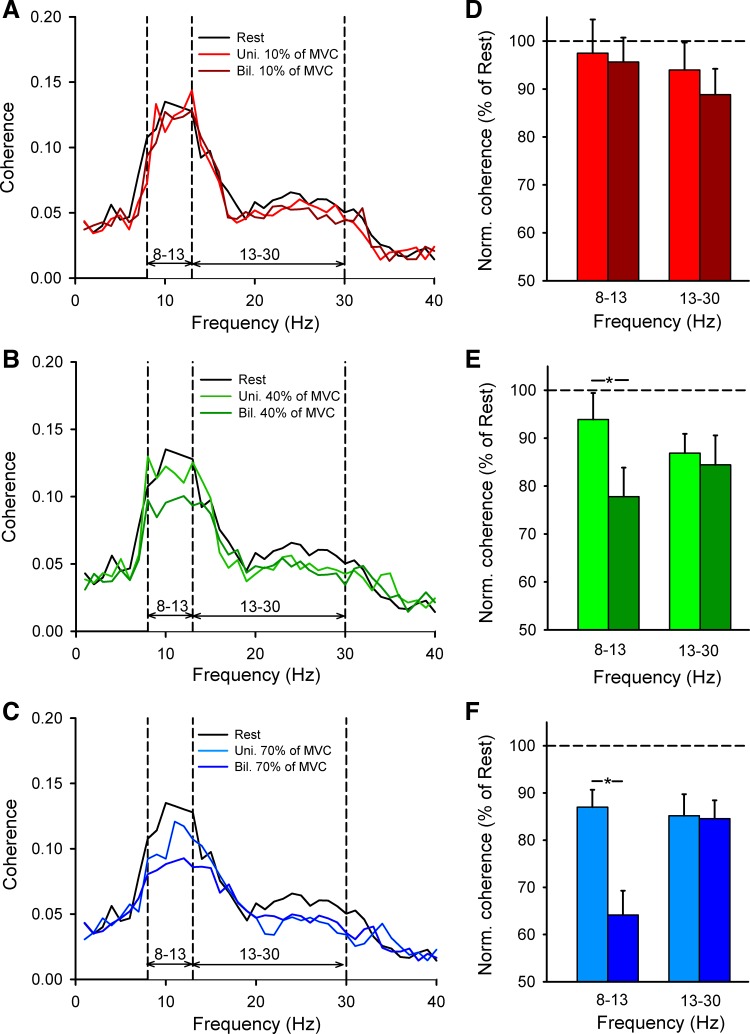
EEG-EEG coherence. *A–C*: EEG-EEG coherence from the dominant to the nondominant motor cortex averaged across 16 subjects performing unilateral (Uni.) and bilateral (Bil.) index finger abduction at 10% (*A*), 40% (*B*), and 70% (*C*) of MVC. *D–F*: normalized mean EEG-EEG coherence in all conditions tested. *x*-Axis shows the frequency band tested (alpha = 8–13 Hz and beta = 13–30 Hz). *y*-Axis shows normalized mean EEG-EEG coherence expressed as % of mean EEG-EEG coherence at each frequency band tested at rest. Error bars indicate SEs. *P < 0.05.

Figure 3, *A–C*, illustrate the population mean EEG-EEG coherence measured from the dominant to nondominant sensorimotor cortex in all subjects tested. Note that coherence in the alpha frequency band decreased to a larger extent during bilateral compared with unilateral contraction at 40% and 70% of MVC, whereas coherence in the beta frequency band remained similar across conditions. See results for EEG-EEG coherence during unilateral contractions in Table 1.

**Table 1.** Coherence and power during unilateral contractions

	Rest	10%	40%	70%	*P*
Coherence					
8–13 Hz	0.13 ± 0.01	0.12 ± 0.01	0.12 ± 0.01	0.10 ± 0.01	<0.01
13–30 Hz	0.067 ± 0.005	0.061 ± 0.004	0.057 ± 0.004	0.056 ± 0.004	<0.001
Power in dominant M1					
8–13 Hz	12.4 ± 0.76	11.2 ± 0.74	10.9 ± 0.83	10.1 ± 0.79	<0.001
13–30 Hz	1.45 ± 0.29	1.21 ± 0.24	1.04 ± 0.17	1.02 ± 0.18	<0.001
Power in nondominant M1					
8–13 Hz	12.2 ± 0.75	11.4 ± 0.84	11.1 ± 0.88	10.5 ± 0.81	<0.001
13–30 Hz	1.31 ± 0.25	1.11 ± 0.11	0.96 ± 0.13	0.94 ± 0.14	<0.001

Values are means ± SD. M1, primary motor cortex.

**Fig. 4.**
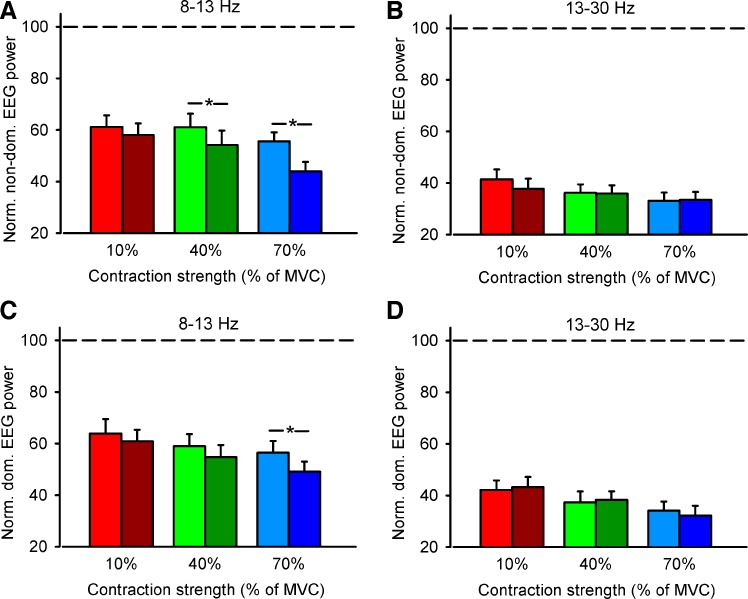
EEG power. Graphs show the normalized mean EEG-EEG power in the left (non-dom., *A* and *B*) and dominant (dom., *C* and *D*) hemisphere in all conditions tested in the alpha (8–13 Hz, *A* and *C*) and beta (13–30 Hz, *B* and *D*) frequency bands. *x*-Axis shows the MVC tested (10%, 40%, and 70% of MVC). *y*-Axis shows normalized mean EEG-EEG power expressed as % of mean EEG-EEG power at each frequency band tested at rest. Horizontal dashed line shows the EEG power at each frequency band at rest. Error bars indicate SEs. *P < 0.05.

Repeated-measures ANOVA showed an effect of FORCE [*F*_(2,15)_ = 12.4, *P* < 0.001], CONDITION [*F*_(1,15)_ = 8.4, *P* = 0.01], and their interaction [*F*_(2,30)_ = 5.1, *P* = 0.01] on normalized EEG-EEG coherence from the dominant to the nondominant hemisphere in the alpha band. Post hoc testing showed a decrease in coherence during bilateral compared with unilateral contraction at 40% (unilateral = 93.8 ± 5.5, bilateral = 77.8 ± 6.0, *P* < 0.001; Fig. 3, *B* and *E*) and 70% (unilateral = 86.9 ± 3.7, bilateral = 64.1 ± 5.1, *P* < 0.001; Fig. 3, *C* and *F*) but not 10% (unilateral = 97.5 ± 7.1, bilateral = 95.6 ± 5.1, *P* = 0.7; Fig. 3, *A* and *D*) of MVC. We found no differences in EEG-EEG coherence in the alpha band at 70% and 40% of MVC was similar (*P* = 0.2). The normalized EEG power decreased to a similar extent during 40% and 70% of MVC (*P* < 0.01; Fig. 4*A*) in the nondominant hemisphere and also decreased during 70% of MVC (*P* < 0.01; Fig. 4*C*) in the dominant hemisphere. In contrast, we found no effect of FORCE [*F*_(2,15)_ = 1.8, *P* = 0.1], TASK [*F*_(1,15)_ = 1.4, *P* = 0.3], or their interaction [*F*_(2,30)_ = 0.3, *P* = 0.7] on normalized EEG-EEG coherence in the beta frequency band (Fig. 3). However, note that EEG-EEG coherence in the beta band decreased to a larger extent during unilateral and bilateral contractions at 40% (*P* < 0.001) and 70% (*P* < 0.001) compared with 10% of MVC without changes in the normalized EEG power.

We also examined EEG-EEG coherence from the nondominant to the dominant hemisphere during the same motor tasks. Repeated-measures ANOVA showed an effect of FORCE [*F*_(2,15)_ = 12.7, *P* < 0.001] but not CONDITION [*F*_(1,15)_ = 1.3, *P* = 0.2] and an effect of their interaction [*F*_(2,30)_ = 3.5, *P* = 0.04] on normalized EEG-EEG coherence in the alpha frequency band. Here coherence decreased during bilateral (10% = 93.3 ± 5.7, 40% = 85.9 ± 5.3, and 70% = 69.9 ± 3.0) compared with unilateral (10% = 94.5 ± 5.1, 40% = 85.9 ± 3.2, and 70% = 83.4 ± 2.9) contraction at 70% (*P* = 0.02) but not at 10% (*P* = 0.6) and 40% (*P* = 0.1) of MVC. Note that, as before, we also found no changes in beta band coherence across force levels [*F*_(2,15)_ = 1.4, *P* = 0.2] and conditions [*F*_(1,15)_ = 3.1, *P* = 0.1]. In addition, we compared directional differences in coherence across hemispheres during increasing levels of MVC in bilateral trials. Repeated-measures ANOVA showed an effect of FORCE [alpha: *F*_(2,15)_ = 38.2, *P* < 0.001; beta: *F*_(2,15)_ = 1.5, *P* = 0.2] but not SIDE [alpha: *F*_(1,15)_ = 0.4, *P* = 0.5; beta: *F*_(1,15)_ = 0.6, *P* = 0.4] or their interaction [alpha: *F*_(2,30)_ = 3.5, *P* = 0.2; beta: *F*_(2,30)_ = 0.2, *P* = 0.8] on normalized EEG-EEG coherence in the alpha and beta frequency bands, suggesting that the magnitude of coherence from the nondominant to dominant and from dominant to nondominant sensorimotor cortex was similar across increasing levels of force.

#### iSP.

**Fig. 5.**
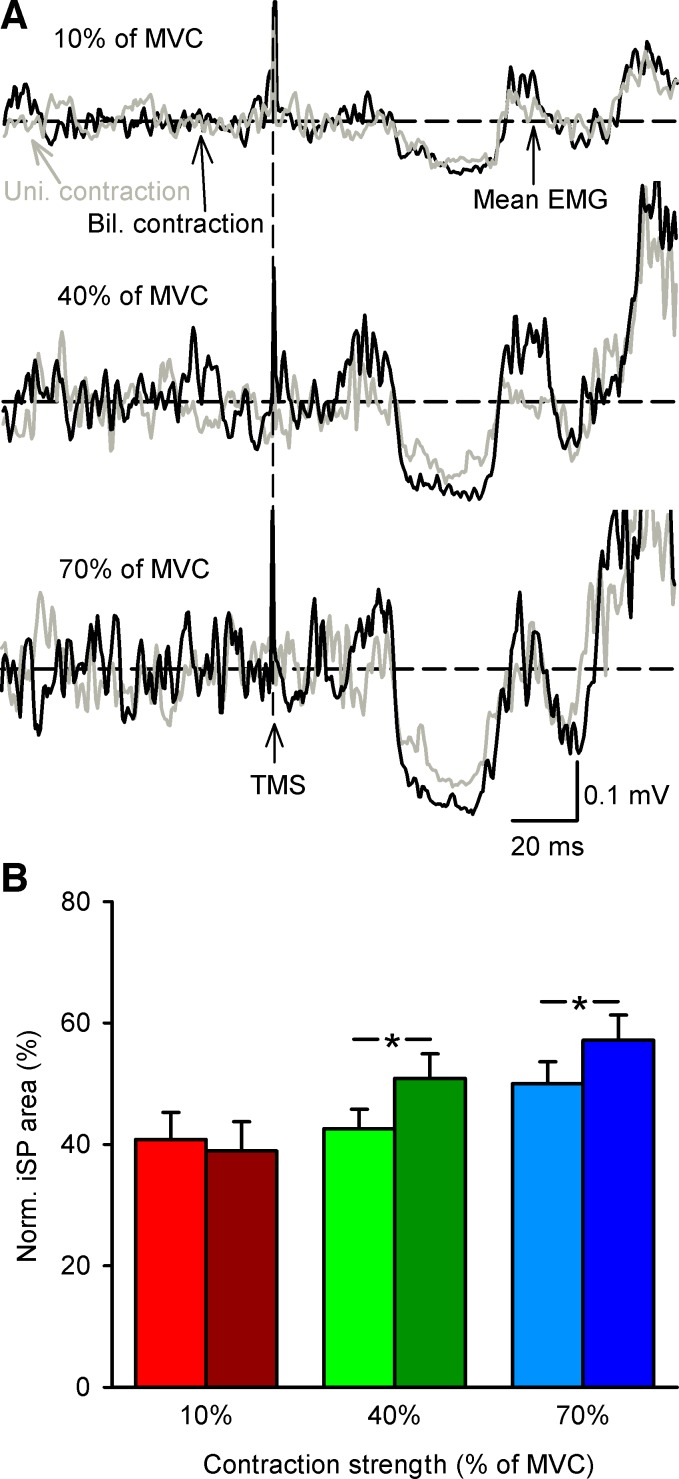
Ipsilateral cortical silent period (iSP). *A*: rectified EMG activity in a representative subject during iSP testing during unilateral (Uni., gray traces) and bilateral (Bil., black traces) index finger abduction at 10%, 40%, and 70% of MVC. Each trace shows the average of 30 trials. Horizontal dashed lines show mean EMG activity over 100 ms before TMS. Vertical dashed lines show the time of TMS during testing. *B*: iSP group data (*n* = 16) during unilateral and bilateral contractions. *x*-Axis shows the force levels tested (10%, 40%, and 70% of MVC). *y*-Axis shows the normalized iSP area. Error bars indicate SEs. *P < 0.05.

Figure 5*A* illustrates examples of the iSP, measured from the dominant to the nondominant motor cortex, elicited in the FDI muscle during unilateral and bilateral contractions in a representative participant. Note that the area of the iSP was increased during bilateral compared with unilateral contraction at 40% and 70% of MVC.

Repeated-measures ANOVA showed an effect of FORCE [*F*_(2,15)_ = 13.8, *P* < 0.001], CONDITION [*F*_(1,15)_ = 8.8, *P* = 0.01], and their interaction [*F*_(2,30)_ = 10.4, *P* < 0.001] on the iSP area (Fig. 5*B*). The iSP area increased during bilateral compared with unilateral contraction at 40% (unilateral = 42.5 ± 12.7%, bilateral = 50.8 ± 16.1%; *P* < 0.001) and 70% (unilateral = 50.0 ± 14.3%, bilateral = 57.1 ± 16.4%; *P* < 0.001) of MVC. No differences were found in the iSP area between 40% and 70% of MVC (*P* = 0.7). When considering individual subjects, 12 of 16 showed an increase in the area of the iSP during bilateral compared with unilateral contraction at 40% and 70% of MVC. No differences were found in the iSP area during unilateral and bilateral 10% of MVC (*P* = 0.4). When the iSP was tested from the nondominant to the dominant motor cortex (but now subjects completed the unilateral task with the dominant hand) we found an effect of FORCE [*F*_(2,6)_ = 27.3, *P* < 0.001], CONDITION [*F*_(1,6)_ = 30.5, *P* = 0.001], and their interaction [*F*_(2,12)_ = 7.2, *P* = 0.009] on the iSP area. As before, the iSP area increased during bilateral compared with unilateral contraction at 40% (unilateral = 36.5 ± 11.1%, bilateral = 41.9 ± 13.2%; *P* < 0.001) and 70% (unilateral = 40.3 ± 12.8%, bilateral = 46.2 ± 11.9%; *P* < 0.001) but not at 10% (unilateral = 35.4 ± 8.4%, bilateral = 37.1 ± 10.5%; *P* = 0.2). Furthermore, we found an effect of FORCE [*F*_(2,6)_ = 17.3, *P* < 0.001] but not SIDE [*F*_(1,6)_ = 0.03, *P* = 0.8] or their interaction [*F*_(2,12)_ = 1.7, *P* = 0.2] on the iSP area.

**Fig. 6.**
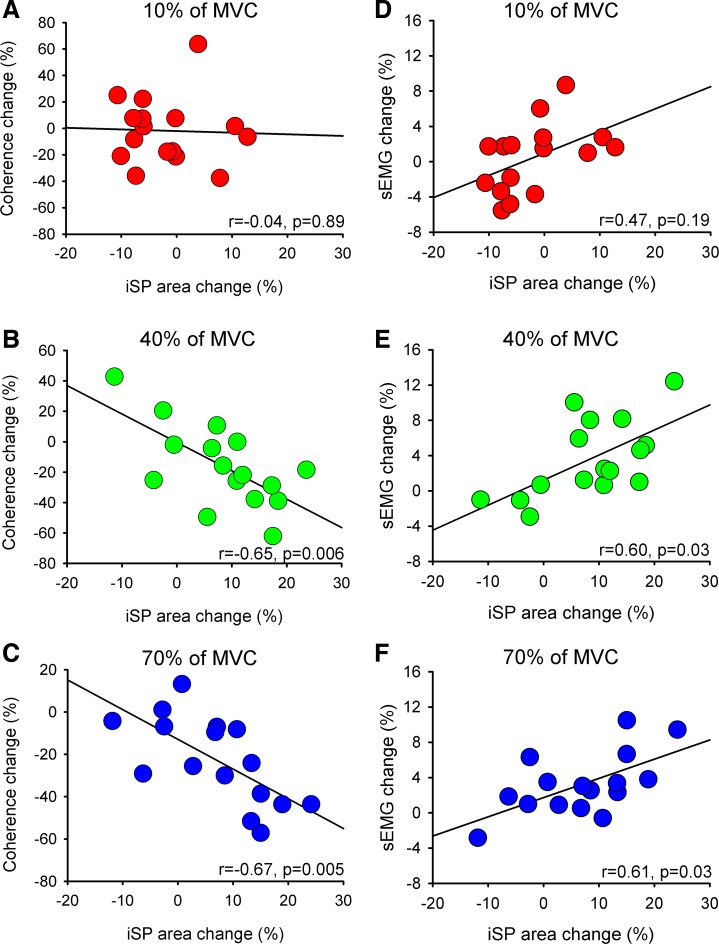
Correlation between EEG-EEG coherence, iSP, and stability index of EMG (sEMG). Graphs show a correlation analysis between changes in EEG-EEG coherence in the alpha frequency band and the iSP area (*A–C*) and between changes in sEMG signals and the iSP area (*D–F*) during bilateral compared with unilateral contractions at 10% (*A* and *D*), 40% (*B* and *E*), and 70% (*C* and *F*) of MVC. In all graphs *x*-axis shows normalized iSP area (difference in the iSP area during bilateral vs. unilateral contractions). *y*-Axis shows normalized EEG-EEG coherence in the alpha frequency band (difference in EEG-EEG coherence during bilateral vs. unilateral contractions, *A–C*) and normalized sEMG (difference in sEMG during bilateral vs. unilateral contractions, *D–F*). *P < 0.05.

A negative correlation was found between changes in the iSP and coherence in the alpha band during bilateral compared with unilateral contractions at 40% (*r* = −0.65, *P* = 0.006; Fig. 6*B*) and 70% (*r* = −0.67, *P* = 0.005; Fig. 6*C*) but not at 10% (*r* = −0.04, *P* = 0.9; Fig. 6*A*) of MVC. Also, a positive correlation was found between changes in the iSP area and measures of motor output steadiness at 40% [sEMG: *r* = 0.60, *P* = 0.03 (Fig. 6*E*); sForce: *r* = 0.61, *P* = 0.03] and 70% [sEMG: *r* = 0.61, *P* = 0.03 (Fig. 6*F*); sForce: *r* = 0.66, *P* = 0.01] but not at 10% [sEMG: *r* = 0.47, *P* = 0.2 (Fig. 6*D*); sForce: *r* = 0.15, *P* = 0.58] of MVC.

#### Effects of peripheral nerve stimulation on EEG-EEG coherence and iSP.

**Fig. 7.**
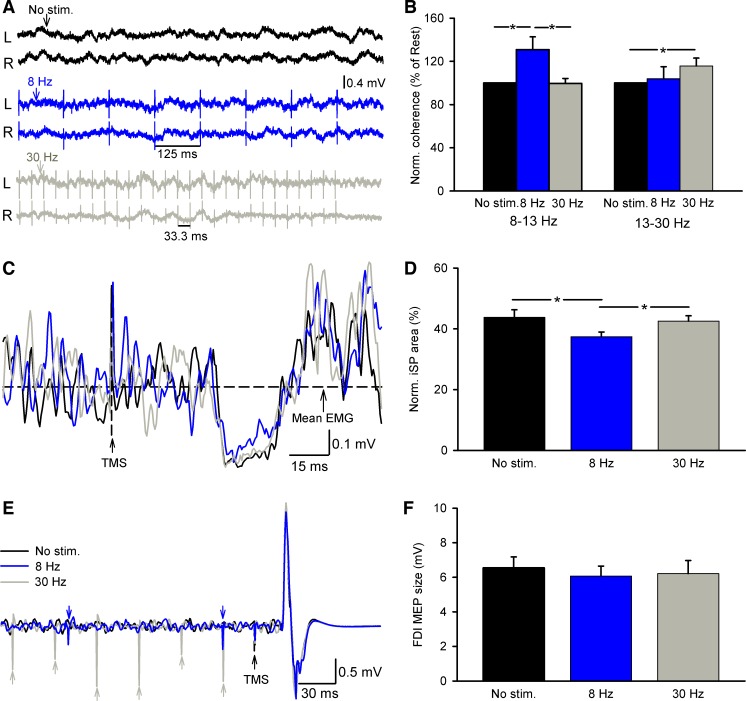
Effects of peripheral nerve stimulation on EEG-EEG coherence and iSP. *A*, *C*, and *E*: EEG signals at rest (*A*) and rectified EMG activity during iSP testing (*C*) and motor evoked potentials (MEPs) in the FDI muscle (*E*) in a representative subject tested during bilateral index finger abduction at 70% of MVC without electrical stimulation (No stim., black traces), with peripheral nerve stimulation of the ulnar nerve at the wrist at 8 Hz (blue traces) and 30 Hz (gray traces). Horizontal dashed line in *C* shows the mean EMG activity over 100 ms before TMS. *B*, *D*, and *F*: group data (*n* = 10). *x*-Axis shows all conditions tested (No stim., 8 Hz, and 30 Hz). *y*-Axis shows the normalized EEG-EEG coherence from the dominant to the nondominant motor cortex in alpha and beta frequency bands (*B*), the normalized iSP area (*D*), and the size of MEPs elicited in the dominant hand during iSP testing (*F*). Note that at rest electrical stimulation at 8 Hz increased EEG-EEG coherence in the alpha frequency band whereas stimulation at 30 Hz increased EEG-EEG coherence in the beta frequency band. Also note that during 70% of MVC stimulation at 8 Hz decreased the iSP area while MEP size elicited by the TMS stimulation was maintained constant across conditions. Error bars indicate SEs. *P < 0.05.

Figure 7*A* illustrates raw traces of EEG signals, the iSP, and MEPs elicited in the FDI muscle during bilateral 70% of MVC in a representative participant. Note that the iSP area decreased during stimulation at 8 Hz compared with 30 Hz and no stimulation.

When tested at rest, repeated-measures ANOVA showed an effect of STIMULATION on the EEG-EEG coherence in the alpha [*F*_(2,4)_ = 28.6, *P* < 0.001] and beta [*F*_(2,4)_ = 7.8, *P* = 0.01] frequency bands (Fig. 7*B*). Here, stimulation at 8 Hz increased coherence in the alpha band by 30.1 ± 10.3% compared with no stimulation without changing coherence in the beta band, whereas stimulation at 30 Hz increased coherence in the beta band by 15.4 ± 5.9% compared with no stimulation without changing coherence in the alpha band. Repeated-measures ANOVA showed an effect of STIMULATION [*F*_(2,9)_ = 9.7, *P* < 0.001; Fig. 7, *C* and *D*; Table 2] on the iSP area during bilateral 70% of MVC. Our results revealed that the iSP area decreased during stimulation at 8 Hz (8 Hz = 37.4 ± 4.7%, 30 Hz = 42.5 ± 5.5%, no stimulation = 43.7 ± 7.8%; *P* = 0.002) but not at 30 Hz compared with no stimulation (*P* = 0.7). No effects of the stimulation at 8 Hz and 30 Hz were found on the FDI MEP size on the dominant FDI during bilateral abduction of 70% of MVC compared with no stimulation [*F*_(2,9)_ = 1.4, *P* = 0.3; Fig. 7, *E* and *F*; Table 2].

**Table 2.** iSP during 70% of MVC

	No Stimulation	8 Hz	30 Hz	*P*
Onset, ms	34.7 ± 2.7	34.2 ± 1.9	33.5 ± 2.9	0.13
Duration, ms	25.4 ± 5.2	26.8 ± 6.8	27.5 ± 5.4	0.39
MEP, mV	6.5 ± 1.9	6.1 ± 1.8	6.2 ± 2.3	0.26
Norm. area, %	43.7 ± 7.8	37.4 ± 4.7	42.5 ± 5.5	<0.001

Values are means ± SD.

iSP, ipsilateral cortical silent period; MVC, maximal isometric voluntary contraction; MEP, motor evoked potential.

## DISCUSSION

Our results demonstrate an inverse relation between alpha oscillations and the iSP during strong levels of bimanual force generation in intact humans. Specifically, we found that EEG-EEG coherence between sensorimotor cortices in the alpha frequency band decreased during bilateral compared with unilateral 40% and 70% but not 10% of MVC, whereas the iSP area increased during bilateral compared with unilateral 40% and 70% but not 10% of MVC. Notably, decreases in coherence in the alpha band were associated with increases in the iSP area during high force levels, and subjects who showed this inverse relation were able to maintain more steady bilateral muscle contractions. Electrical stimulation of the ulnar nerve at the wrist at the alpha frequency increased coherence in the alpha band and decreased the iSP area during 70% of MVC. We propose that inverse interactions between neural pathways mediating alpha oscillatory activity and transcallosal inhibition between motor cortices might contribute to the steadiness of strong bilateral isometric muscle contractions.

### 

#### Interhemispheric communication during bimanual force generation.

It is well accepted that interactions between motor cortices during bimanual force generation take place, at least in part, through the corpus callosum (Carson 2005; Diedrichsen et al. 2003; Giovannelli et al. 2009; Perez et al. 2014; Soteropoulos and Perez 2011; Tazoe et al. 2013; Yedimenko and Perez 2010). Thus we measured EEG-EEG coherence between sensorimotor cortices to examine interhemispheric communication (Andrew and Pfurtscheller 1996; Serrien et al. 2003, 2004) during increasing levels of bimanual force. Our findings that EEG-EEG coherence decreased during bilateral compared with unilateral force at 40% and 70% of MVC in the alpha but not the beta frequency band agree with evidence suggesting that interhemispheric interactions at these frequency bands serve distinct functions (Brinkman et al. 2014) and are related to separate functional networks (Hari and Salmelin 1997). This also agrees with previous studies showing that coherence between sensorimotor cortices in the alpha band is sensitive to detect changes during strong levels of force generation (Abdul-latif et al. 2004; Svoboda et al. 2002). The decrease in coherence in the alpha but not the beta band might be related to a lesser synchronization between motor cortical networks during strong voluntary contractions (Kristeva et al. 2007; Perez et al. 2012). This is supported by the decrease in EEG spectral power that we observed in the alpha but not the beta band in both hemispheres during high force levels. Since the beta rhythm is associated with motor cortical function (Baker 2007; Brown 2000), it is intriguing that beta coherence did not change during increasing levels of bilateral compared with unilateral force. However, it is important to consider that most associations of the beta rhythm with motor cortical function have been demonstrated for EEG-EMG coherence (Brown 2000). Indeed, some differences have been reported between EEG-EEG and EEG-EMG coherence at these different frequency bands. For example, alpha and beta EEG oscillations are largely detected from the hand post-Rolandic somatosensory area and the pre-Rolandic motor area, respectively (Pfurtscheller and Lopes Da Silva 1999), whereas EEG-EMG coherence is usually absent in the alpha (Baker et al. 2003) and present in the beta (Baker 2007) frequency band when electrodes are positioned at similar locations. Although the magnitude of beta band coherence was similar during unilateral and bilateral increasing force levels, the overall magnitude of beta band coherence decreased during 40% and 70% compared with 10% of MVC. This is consistent with previous evidence showing a progressive reduction in beta band coherence in the sensorimotor cortex contralateral to a hand performing increasing levels of force (Perez et al. 2012) and agrees with evidence showing that EEG-EEG coherence in the beta band changes with increasing task demands (Serrien et al. 2004).

We also measured the iSP to examine interhemispheric communication between motor cortices during increasing levels of bimanual force. It is thought that the iSP measures transcallosal inhibition from the stimulated to the contralateral motor cortex (Ferbert et al. 1992; Meyer et al. 1995; Trompetto et al. 2004), and a transcallosal route of the iSP has been supported by an absent or delayed iSP in patients with agenesis or surgical lesions of the corpus callosum (Meyer et al. 1995). We found that the iSP area increased during bilateral compared with unilateral forces at 40% and 70% of MVC. This agrees with findings showing that the magnitude of iSP increased during bilateral compared with unilateral voluntary contractions (Giovannelli et al. 2009; Perez et al. 2014; Soteropoulos and Perez 2011; Yedimenko and Perez 2010). We also found that the iSP remained similar during unilateral and bilateral contractions at 10% of MVC in agreement with previous results (Fling and Seidler 2012). Indeed, some studies reported that stronger contractions by one arm are needed to detect differences in the magnitude of the iSP during bilateral forces (Perez et al. 2014; Soteropoulos and Perez 2011; Yedimenko and Perez 2010). Altogether, our results show an inverse modulation of alpha oscillations and transcallosal inhibition between motor cortices during strong levels of bilateral isometric muscle contractions.

An intriguing question is whether changes in coherence in the alpha frequency band and the iSP interact, at least to some extent, during strong levels of bilateral isometric force. Some of our results support this possibility. First, we found that changes in the iSP area were negatively correlated with changes in coherence in the alpha band at 40% and 70% of MVC. The correlations found between EEG-EEG coherence in the alpha frequency band and the iSP at stronger levels of force suggest that changes in EEG-EEG coherence reflect changes in cortical interactions. It has been shown that TMS has direct access to the circuitry in the motor cortex involved in the generation of oscillations of corticospinal cells (Hansen and Nielsen 2004). Thus our results, as previous findings (Baker and Baker 2003), indicate that inhibitory cortical circuits might have an effect on the modulation of cortical oscillations. Second, we found that both the magnitude of the iSP and EEG-EEG coherence in the alpha band remained similar during 40% and 70% of MVC. Evidence showed that the firing rate of motor cortical cells (Evarts et al. 1983; Maier et al. 1993) and BOLD signal from motor cortex (Dettmers et al. 1995) saturated at high force levels. Thus a lack of modulation in both measurements at similar levels of force might be in part related to a ceiling effect during bilateral contractions, suggesting that these processes might undergo parallel changes. The lack of changes in EEG-EEG coherence at both frequencies during bilateral compared with unilateral 10% of MVC agrees with previous findings showing no changes in coherence (Andres et al. 1999; Serrien et al. 2004) and oscillatory activity (Murthy and Fetz 1996) between sensorimotor cortices at similar low force levels. Third, we found that the iSP area measured during bilateral 70% of MVC increased with electrical stimulation at 8 Hz but not at 30 Hz. We used electrical stimulation of afferent fibers as a source modulator since alpha oscillations (Budini et al. 2014) and interhemispheric inhibition measured by TMS (Tsutsumi et al. 2012) are changed by similar peripheral afferent inputs. The fact that the same stimulation paradigm increased alpha coherence and decreased the iSP also supports the view of possible interactions between neural pathways mediating these effects. It is important to consider that coherence between EEG channels spaced <10–12 cm apart might contain contributions due to volume conduction (Mima and Hallett 1999; Nunez et al. 1997). Although in our study to decrease this effect EEG signals were recorded with bipolar electrodes 4 cm apart (Nunez et al. 1997) and EEG-EEG coherence was absent at lower (1–2 Hz) and higher (500–510 Hz) frequencies (Winter et al. 2007), we cannot completely exclude the possibility that changes in volume conduction affected our results.

#### Functional significance.

Despite the undoubted importance of callosal pathways in interhemispheric communication, their functional role during bimanual force generation remains largely unknown. A possibility is that strong transcallosal inhibition between motor cortices could represent a mechanism to suppress neural cross talk (Rokni et al. 2003) in EEG signals when executing simultaneous movements with both hands. Another possibility is that during strong bilateral forces more motoneurons are active and an increase in the iSP could help to prevent unwanted muscle activity to match the desired level of effort. On one hand, studies have proposed that changes in oscillations in the alpha frequency band might have a role in error corrections (Mehrkanoon et al. 2014) and suppressing task-irrelevant neuronal processing (Haegens et al. 2011). On the other hand, it has been proposed that changes in interhemispheric inhibition between motor cortices might contribute to suppress unwanted EMG activity (Cincotta and Ziemann 2008) and to suppress task-irrelevant activity during specific types of finger movements (Tazoe and Perez 2013). Thus it is tempting to speculate that both measurements could be linked in a functional manner since subjects who showed decreases in coherence in the alpha band and increases in the iSP were able to maintain more steady strong muscle contractions. However, caution must be taken in extrapolating these results to bilateral functions since correlations do not imply causality and previous evidence showed that interactions between actively moving arms and those obtained during isometric contractions differ (Carson 1995; Carson et al. 1994). Regardless of the interpretation of these results, the lack of directional differences in the magnitude of alpha band coherence and the iSP in our study favors the view that these mechanisms might contribute to general aspects of bimanual force generation.

## GRANTS

This work was supported by National Institute of Neurological Disorders and Stroke Grants R01 NS-076589 and NS-0900622, the Department of Veterans Affairs Grant 3397626, and the Medical Research Council (United Kingdom; D. S. Soteropoulos).

## DISCLOSURES

No conflicts of interest, financial or otherwise, are declared by the author(s).

## AUTHOR CONTRIBUTIONS

Author contributions: J.L., T.T., D.S.S., and M.A.P. conception and design of research; J.L., T.T., D.S.S., and M.A.P. performed experiments; J.L., T.T., D.S.S., and M.A.P. analyzed data; J.L., T.T., D.S.S., and M.A.P. interpreted results of experiments; J.L., T.T., D.S.S., and M.A.P. prepared figures; J.L., T.T., D.S.S., and M.A.P. drafted manuscript; J.L., T.T., D.S.S., and M.A.P. edited and revised manuscript; J.L., T.T., D.S.S., and M.A.P. approved final version of manuscript.
